# Supplementary material for: Predicting visual function by interpreting a neuronal wiring diagram
Source: Nature. 2024 Oct 2;634(8032):113–23. doi: 10.1038/s41586-024-07953-5 (PMC11446822; doi:10.1038/s41586-024-07953-5)
Supplement: Supplementary file 6 — CRF and ERF predictions for individual TmY4 and TmY9 cells. Analogous to Supplementary Data 3, but for TmY target types. Shown are the top four monosynaptic pathways, the strongest pathway passing through each of the top ten intermediary types (ranking from Extended Data Fig. 7), and the trisynaptic pathway Tm1–TmY–Dm3–TmY (see the section entitled Prediction of spatial normalization). [file 41586_2024_7953_MOESM6_ESM.zip › DataS4/TmY9q/720575940640404021.pdf]

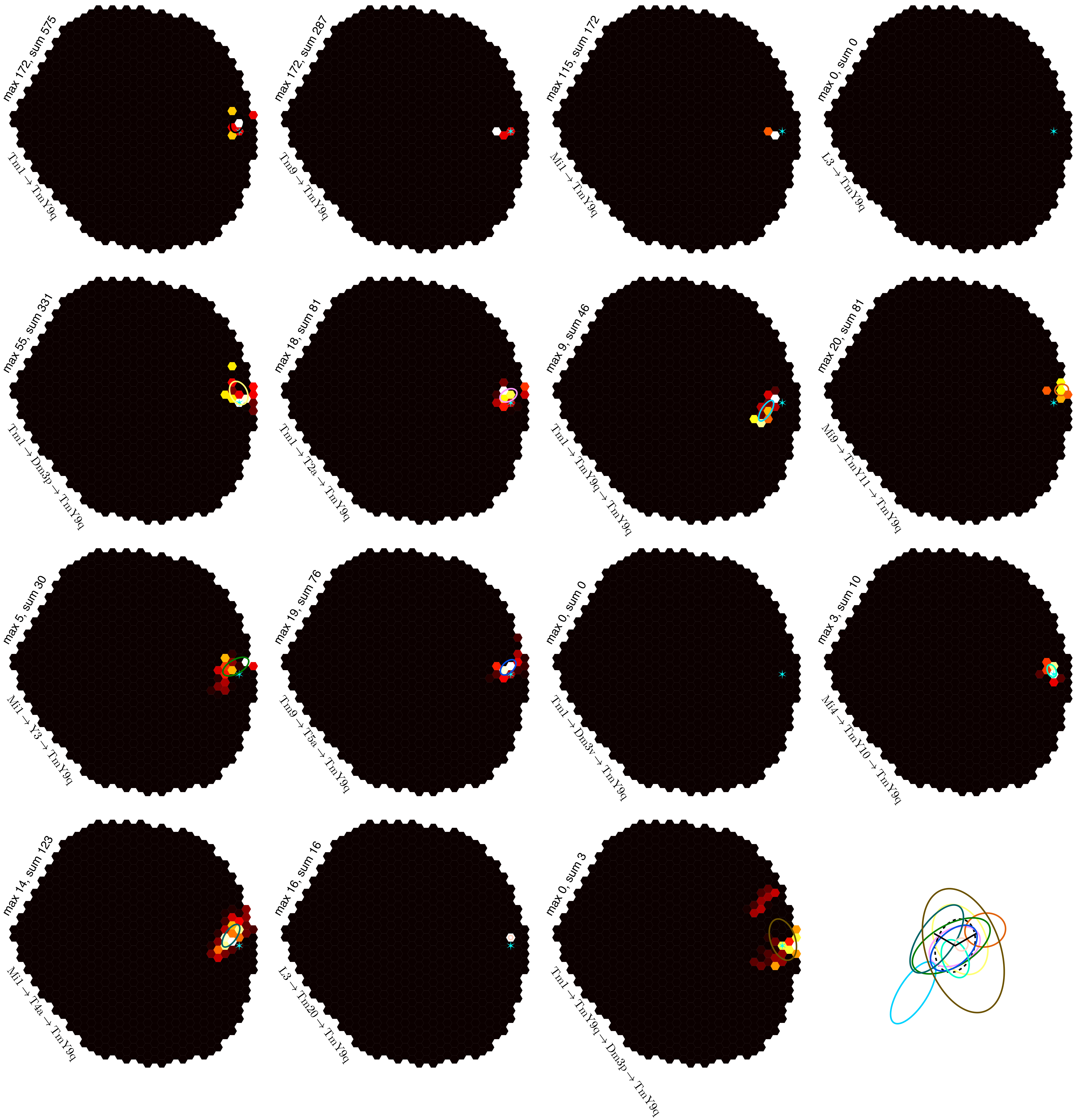

max 172, sum 575
Tm1 → TmY9q
max 172, sum 287
Tm9 → TmY9q
max 115, sum 172
Mi1 → TmY9q
max 0, sum 0
L3 → TmY9q
max 55, sum 331
Tm1 → Dm3p → TmY9q
max 18, sum 81
Tm1 → T2a → TmY9q
max 9, sum 46
Tm1 → TmY9q → TmY9q
max 20, sum 81
Mi9 → TmY11 → TmY9q
max 5, sum 30
Mi1 → Y3 → TmY9q
max 19, sum 76
Tm9 → T5a → TmY9q
max 0, sum 0
Tm1 → Dm3v → TmY9q
max 3, sum 10
Mi4 → TmY10 → TmY9q
max 14, sum 123
Mi1 → T4a → TmY9q
max 16, sum 16
L3 → Tm20 → TmY9q
max 0, sum 3
Tm1 → TmY9q → Dm3p → TmY9q
